# Treatment effects of lysozyme-shelled microbubbles and ultrasound in inflammatory skin disease

**DOI:** 10.1038/srep41325

**Published:** 2017-01-24

**Authors:** Ai-Ho Liao, Chi-Ray Hung, Chieh-Fu Lin, Yi-Chun Lin, Hang-Kang Chen

**Affiliations:** 1 Graduate Institute of Biomedical Engineering, National Taiwan University of Science and Technology, Taipei 10607, Taiwan; 2 Department of Biomedical Engineering, National Defense Medical Center, Taipei 11490, Taiwan; 3 Graduate Institute of Manufacturing Technology, National Taipei University of Technology, Taipei 10608, Taiwan; 4 Graduate Institute of Medical Sciences, National Defense Medical Center, Taipei 11490, Taiwan

## Abstract

Acne vulgaris is the most common skin disorder, and is caused by *Propionibacterium acnes (P. acnes*) and can induce inflammation. Antibiotic therapy often needs to be administered for long durations in acne therapy, which results in extensive antibiotic exposure. The present study investigated a new treatment model for evaluating the antibacterial effects of lysozyme (LY)-shelled microbubbles (MBs) and ultrasound (US)-mediated LY-shelled MBs cavitation against *P. acnes* both *in vitro* and *in vivo*, with the aims of reducing the dose and treatment duration and improving the prognosis of acne vulgaris. In terms of the *in vitro* treatment efficacy, the growth of *P. acnes* was inhibited by 86.08 ± 2.99% in the LY-shelled MBs group and by 57.74 ± 3.09% in the LY solution group. For US power densities of 1, 2, and 3 W/cm^2^ in the LY-shelled MBs group, the growth of *P. acnes* was inhibited by 95.79 ± 3.30%, 97.99 ± 1.16%, and 98.69 ± 1.13%, respectively. The *in vivo* results showed that the recovery rate on day 13 was higher in the US group with LY-shelled MBs (97.8 ± 19.8%) than in the LY-shelled MBs group (90.3 ± 23.3%). Our results show that combined treatments of US and LY-shelled MBs can significantly reduce the treatment duration and inhibit *P.-acnes*-induced inflammatory skin diseases.

According to the Global Burden of Disease study, acne vulgaris is the most common cutaneous condition, affecting ~85% of young adults aged 12–25 years^1^. The goals of treatments for acne vulgaris are to inhibit comedone formation using retinoids and to suppress *Propionibacterium acnes (P. acnes*) using antibiotics. Combination therapy of topical antibiotics and either benzoyl peroxide or topical retinoids is more effective for limiting the increasing resistance to antibacterial therapy than either agent used alone^2^,^3^,^4^. However, retinoid monotherapy, retinoid in combination with benzoyl peroxide, or antibiotic/benzoyl peroxide combinations are not ideal for maintenance therapy^4^. Some common antibiotics exhibit minimal systemic absorption and may be responsible for the development of bacterial resistance^5^,^6^,^7^,^8^. The present study applied lysozyme (LY), an alternative antibiotic, with microbubbles (MBs), and combined them with ultrasound (US) with the aims of reducing the dose and treatment duration and improving the prognosis of acne vulgaris while also avoiding bacterial resistance.

Based on the characteristics of nasal secretions, LY was found to suppress bacterial growth by Alexander Fleming in 1922, before the discovery of penicillin^9^. LY is a naturally occurring enzyme found in bodily secretions such as tears, saliva, and milk, and is considered a part of the innate immune system in most mammals^10^. LY degrades peptidoglycan in the bacterial cell wall, which leads to cell death^11^. Although LY is not a typical agent for acne treatment, LY is a safe adjunct to antifungals and could be used to improve acne treatment due to its antibactericidal effect^12^. Incubation of *P. acnes* supernatant with LY at various concentrations reduced the *P. acnes* activity, and LY-triclosan complexes were found to significantly enhance bactericidal activity against several strains of Gram-positive and Gram-negative bacteria^13^. These results indicate that the membrane-disrupting function of LY can be utilized to specifically target antimicrobial drug(s) at pathogen cells and heralds a fascinating opportunity for the potential of LY-triclosan complexes as novel antimicrobial strategy for human therapies^13^.

Stable air-filled LY-shelled MBs were recently synthesized using high-intensity US-induced emulsification of partly reduced LY in aqueous solutions^14^. That study investigated the possibility of using LY-shelled MBs for delivering proteins and nucleic acids in prophylactic and therapeutic applications. MBs are small gas-filled colloidal particles that are commonly applied in clinical applications as contrast agents for US imaging via intravenous injection. The shell of MBs is primarily based on protein, polymer, or lipid coatings. Our previous studies have demonstrated different conditions of albumin-shelled MBs for enhancing their penetration in transdermal delivery *in vivo*^15^,^16^,^17^. Combined treatment with US and MBs can increase skin permeability and enhance α-arbutin delivery to inhibit melanogenesis without damaging the skin in mice^15^. Combining US with MBs of different sizes can produce different degrees of skin permeability so as to enhance the delivery of high-molecular-weight drugs^16^. Moreover, the efficacy of applying US and MBs with agarose gel at different concentrations for enhancing the skin permeability has been demonstrated, which is due to the viscosity of the agarose increasing with its concentration^17^. The use of US plus MBs also can increase the skin permeability and thereby enhance the delivery of diclofenac sodium gel to inhibit inflammation of the tissues surrounding an arthritic ankle^18^. A new type of US-contrast-agent/albumin-shelled MB was recently created that absorb chitosan oligosaccharide lactate and minoxidil, and combining these MBs with sonication by US energy in the water phase enhanced hair growth while shortening the treatment period^19^. However, the stability of protein-shelled MBs was significantly greater for those shelled with LY than with albumin^13^. Moreover, LY can used to treat *P. acnes* due to its antibactericidal effect. Therefore, the present study applied LY as the shell of MBs and combined them with US with the aim of reducing the dose and treatment duration and improving the prognosis of acne vulgaris.

## Materials and Methods

### Preparation characterization of LY-shelled MBs

In accordance with a typical synthesis procedure, 50 mg of chicken egg-white LY was dissolved in 1 ml of 50 mM Tris buffer (pH 8), and then 20 mg of reducing agent (DL-DTT) was added and the solution was shaked at 50 rpm for 15 min at room temperature to allow sufficient time for partial reduction to occur. MBs were generated by sonicating this solution in perfluoropropane (C_3_F_8_) gas using a sonicator at powers of 80, 120, and 180 W (Branson Ultrasonics, Danbury, CT, USA) for 30 s. The MBs were centrifuged at 1200 rpm (128.6 × *g*) for 2 min and then washed three times to eliminate the Tris buffer and DL-DTT using an Milli-Q water (pH = 6.4, resistance = 18.2 mΩ). The number of LY-shelled MBs in the solution was measured with the MultiSizer III device (Beckman Coulter, Fullerton, CA, USA) using a 30-μm aperture probe whose measurement boundary ranged from 0.6 to 20 μm. The size distribution in the suspension was measured by dynamic light scattering (Nanoparticle Analyzer, Horiba, Kyoto, Japan). The containing of LY in original LY-shelled MBs solution was measured using a UV spectrometer (Lambda 40 UV/VIS Spectrometer, Perkin Elmer, Norwalk, CT, USA) at 280 nm. To characterize the morphology of LY-shelled MBs, the LY-shelled MBs were filtered with a 5-μm syringe filter (Sartorius, Goettingen, Germany) and then hardened using 0.25% glutaraldehyde (Sigma-Aldrich, St. Louis, IL, USA). The morphology of the hardened LY-shelled MBs was studied using scanning electron microscopy (SEM) after coating the samples with platinum (achieved using 20 mA for 20 min) using an automatic sputter coater (JFC-1300, JEOL, Tokyo, Japan). SEM images were recorded at an accelerating voltage of 15 kV.

### Microorganism cultures

*P. acnes* (BCRC10723, Bioresource Collection and Research Center, Hsinchu, Taiwan) was cultured on Reinforced Clostridium Medium (RCM, Sigma-Aldrich) under anaerobic conditions using an Anaero Pack (Mitsubishi Gas Chemical Company, Tokyo, Japan) at 37 °C. To keep the bacterial survival and growth stable, 50  μl of *P. acnes* (2 × 10^7^ colony-forming units [CFU]/ml) was added to 3 ml of RCM (1.9 g/50 ml, Sigma-Aldrich) in a sterilized test tube (14-ml polypropylene round-bottomed tube, BD Falcon™, Sparks, MD, USA).

### *In vitro* antimicrobial efficacy of LY-shelled MBs against P. acnes under different conditions

For the antigrowth assay, *P. acnes* solutions were treated with 1%, 5%, and 10% LY-shelled MBs (using a sonicator at powers of 120 W, containing 0.25, 0.75, and 2.5 mg/ml LY) without and with US at power densities of 1, 2, and 3 W/cm^2^ for 1 min. The US probe of the sonoporation gene transfection system (ST 2000 V, NepaGene, Ichikawa, Japan) was placed 5 mm under the surface of the solutions. Before the experiments, the concentration of *P. acnes* was measured using a UV spectrometer (Lambda 40 UV/VIS Spectrometer, Perkin Elmer, Norwalk, CT, USA) at 600 nm. *P. acnes* solutions were then harvested by centrifugation (Allegra 21 R centrifuge, Beckman Coulter) at 10,537 × *g* for 1 min, washed three times with Milli-Q water, and then suspended in Milli-Q water. *P. acnes* samples (2 × 10^7^ CFU/ml) were withdrawn and incubated with 500 μl of LY-shelled MBs at various concentrations at room temperature with shaking at 20 rpm for 30 min. The *P.-acnes*-containing LY-shelled MBs solutions were then centrifuged (1200 rpm, 128.6× *g*) for 2 min, washed three times to eliminate the LY-shelled MBs, and the concentration of *P. acnes* was measured. The antibacterial effects were quantified using the following equation^20^:


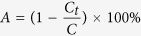


where *C* and *C*_*t*_ are the concentrations of *P. acnes* before and after treatment, respectively.

### *In vitro* treatment efficacy of LY-shelled MBs against P. acnes colonies

*P. acnes* was adjusted to a concentration to 2 × 10^7^ CFU/ml using the plate count method, mixed with 5% LY-shelled MBs (8.4 × 10^6^ bubbles/ml, containing 0.75 mg/ml LY) in an Eppendorf tube, and sonicated by the 1-MHz US transducer of the sonoporation system successively at the following acoustic power densities: 1 W/cm^2^ for 1 min, 2 W/cm^2^ for 1 min, and 3 W/cm^2^ for 1 min. The duty cycle was set at 50% and a 0.6-cm-diameter US transducer was used. The change in temperature during US sonication at power densities of 2 and 3 W/cm^2^ for 1 min at 37 °C did not exceed 0.3 °C, as measured by a thermometer (Optris LS, Optris, Berlin, Germany). The solution was rested for 30 min, and then samples were diluted 1:10^4^ in PBS, and 10 μL of each sample was spotted on RCM agar plates. The samples were incubated at 37 °C under anaerobic conditions for 3 days, and then the CFU of *P. acnes* were quantified with the aid of image-analysis software (ImageJ, National Institutes of Health, Bethesda, MD, USA).

### Animal treatments

**Figure 1:**
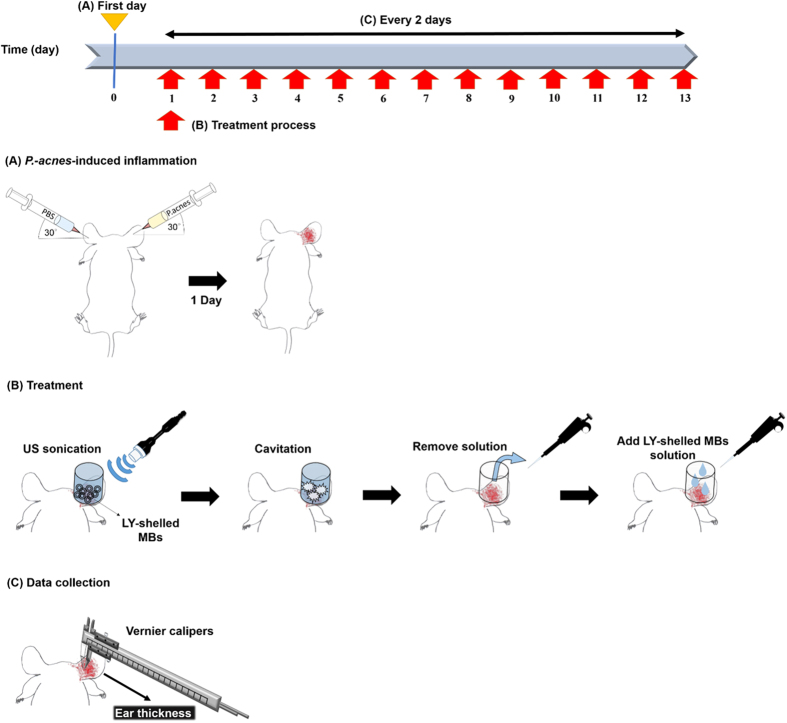
Schematic drawing (not to scale) of the experimental procedure of the animal treatments.

A schematic diagram of the experimental procedure of animal treatments is shown in Fig. 1. Eight-week-old ICR mice weighing 20–25 g were obtained from Bio Lasco (Taipei, Taiwan). The experimental protocol was approved by the Institutional Animal Care and Use Committee of the National Defense Medical Center, Taipei, Taiwan. Animals were cared for in compliance with institutional guidelines and regulations. Throughout the experiments, the animals were housed in stainless-steel cages in an air-conditioned room with the temperature maintained at 25–28 °C and with alternating light and dark periods of 12 hours each. The animals were acclimatized for 7 days prior to the experiments. *P.-acnes*-induced inflammation was induced according to a previously reported procedure^21^,^22^. Aliquots (20 μl) of living *P. acnes* (1 × 10^7^ CFU) suspended in PBS (pH = 7.4) were injected intradermally into the central portion of the right ear. As a control, 20 ml of PBS was injected into the left ears of the same mice.

The animal daily treatments were performed for 13 days, beginning after 1 day. The animals were divided into the following three groups (*n* = 5 per group, treatment applied once daily for 3 weeks): (i) no treatment (Group C), (ii) penetrating LY-shelled MBs alone (Group M), and (iii) US with LY-shelled MBs (Group MU). The US was applied at 3 W/cm^2^ (acoustic pressure = 0.266 MPa) for 1 min, and 0.75 mg/ml (1 ml/cm^2^) LY-shelled MBs were used in all cases. The US probe (1.2 cm in diameter) was placed 5 mm from the ear. A round area with a radius of 1.2 cm and a height of 5 mm on each ear was encircled with US gel to prevent leakage and loaded with LY-shelled MBs. The increase in ear thickness was measured using microcalipers and calculated for the *P.-acnes*-challenged ear as a percentage of a PBS-injected control.

### Histochemistry

Ear tissue samples that included the treatment area were cut immediately after the experiments and stored in a 4% formalin solution. For histological observations, the ear was cross-sectioned, stained with hematoxylin and eosin (Sigma-Aldrich), and viewed under a microscope (Zeiss Primo Star, Zeiss-Jena, Jena, Germany).

### Statistical analysis

The obtained data were analyzed statistically using Student’s *t*-test. Different groups were compared using one-way ANOVA followed by Tukey’s multiple-comparison test. A probability value of *p* < 0.05 was considered indicative of a significant difference. Data are presented as mean ± SD values.

## Results

### Characterization of LY-shelled MBs

**Figure 2:**
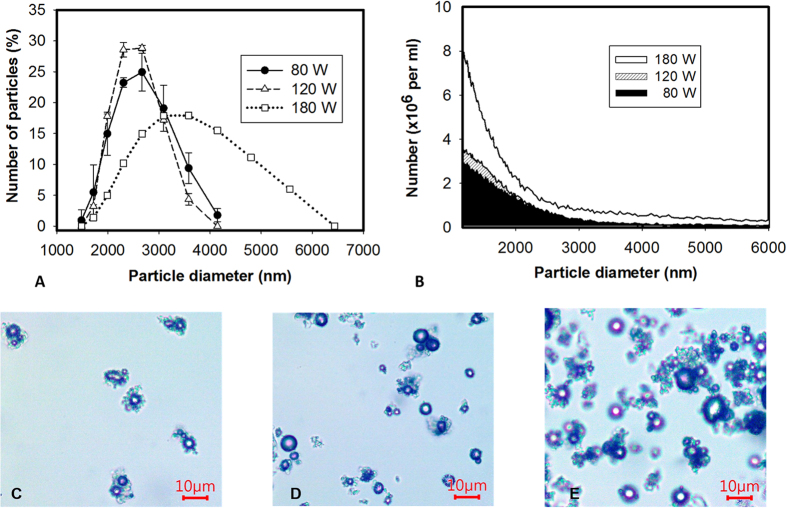
Size (**A**) and concentration (**B**) distributions of the LY-shelled MBs constructed using sonicator powers of 80, 120, and 180 W in suspensions measured by dynamic light scattering and electrical sensing zone, respectively. Microscope images (**C–E**) of the LY-shelled MBs constructed using sonicator powers of 80 (**C**), 120 (**D**), and 180 (**E**) W in suspensions.

**Figure 3:**
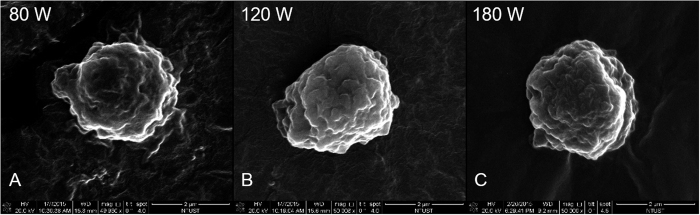
SEM images of the LY-shelled MBs constructed using sonicator powers of (**A**) 80 W, (**B**) 120 W, and (**C**) 180 W.

The diameters of LY-shelled MBs constructed using the sonicator at powers of 80, 120, and 180 W were 2586 ± 211, 2473 ± 124, and 2866 ± 135 nm, respectively (Fig. 2A, n = 8); the corresponding concentrations of LY-shelled MBs were 1.47 ± 0.04 × 10^8^/ml, 1.68 ± 0.02 × 10^8^/ml, and 3.26 ± 0.04 × 10^8^/ml (Fig. 2B). Figure 2A,C,D and E indicate that the size distribution did not vary with the sonicator power, but LY-shelled MBs made using the sonicator at 80 and 120 W had a narrow size distribution ranging from 1500–4200 nm in diameter. Figure 3A–C show SEM images of LY-shelled MBs constructed using the sonicator at powers of 80, 120, and 180 W, respectively. The composite structures of the LY-shelled MBs for different sonicator powers were revealed by SEM, indicating small protein particles on the LY-shelled MB surface with no evident differences for different US powers.

### *In vitro* antimicrobial efficacy of LY-shelled MBs against P. acnes under different conditions

**Figure 4:**
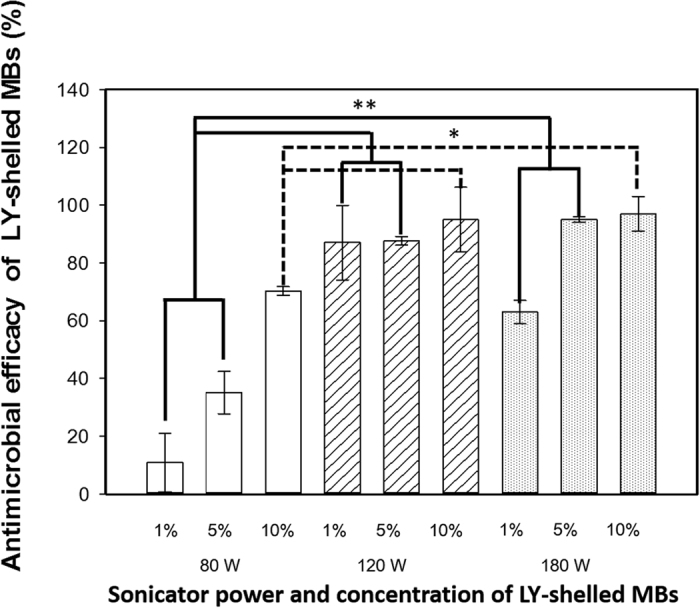
Antimicrobial efficacy of LY-shelled MBs against *P. acnes* under different conditions (*p < 0.05, **p < 0.01). Data are mean and SD values.

Figure 4 shows the antimicrobial efficacies of 1%, 5%, and 10% LY-shelled MBs constructed using the sonicator at powers of 80, 120, and 180 W (n = 6). The antimicrobial efficacies of LY-shelled MBs constructed using sonicator powers of 120 and 180 W were significant higher than for 80 W at each concentration (*p* < 0.05). The antimicrobial efficacy of 5% LY-shelled MBs constructed using sonicator powers of 120 W (87.6 ± 1.5%) and 180 W (95.1 ± 1.2%) were both close to 90%, but 120-W LY-shelled MBs had a more uniform distribution. Although the antimicrobial efficacy did not differ significantly among 1%, 5%, and 10% LY-shelled MBs constructed using a sonicator power of 120 W (*p* > 0.05), the variability of antimicrobial efficacy was more obvious for 1% and 10% LY-shelled MBs than for 5% LY-shelled MBs. Therefore, 5% LY-shelled MBs constructed using a sonicator power of 120 W was selected for use in all of the subsequent experiments performed in this study. The containing of LY in original LY-shelled MBs solution constructed using a sonicator power of 120 W is 25 mg/ml.

### *In vitro* treatment efficacy of LY-shelled MBs combined with US against P. acnes colonies

**Figure 5:**
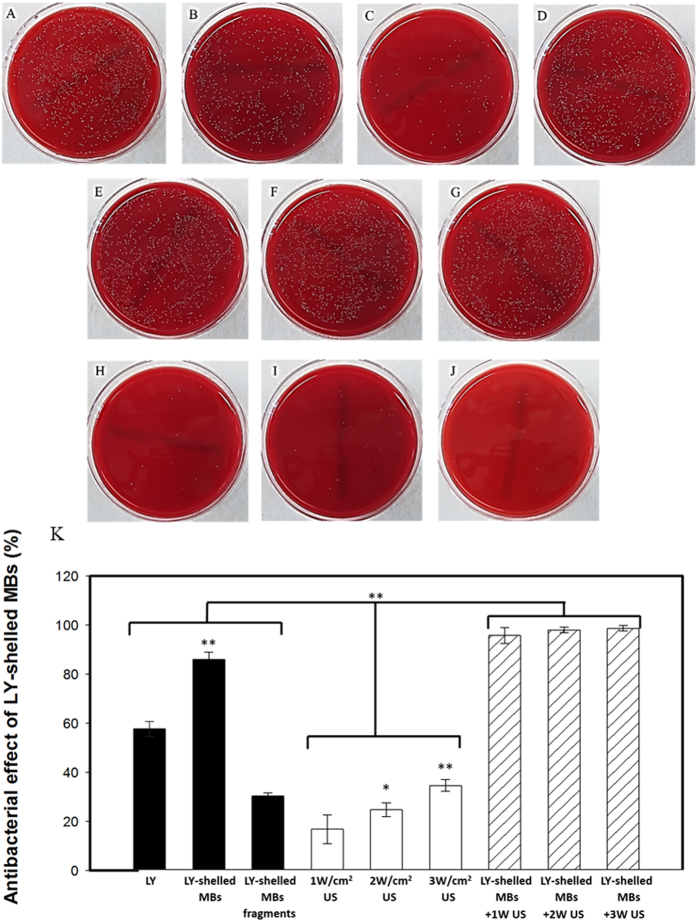
Photographs of (**A**) control, (**B**) LY solution, (**C**) LY-shelled MBs, (**D**) LY-shelled MBs fragments, (**E**) 1 W/cm^2^ US, (**F**) 2 W/cm^2^ US, (**G**) 3 W/cm^2^ US, (**H**) 1 W/cm^2^ US with LY-shelled MBs, (**I**) 2 W/cm^2^ US with LY-shelled MBs, (**J**) 3 W/cm^2^ US with LY-shelled MBs, and (**K**) quantitative results of the treatment efficacy in various treatment groups against *P. acnes* colonies (*n* = 5) (*p < 0.05, **p < 0.01). Data are mean and SD values.

Figure 5 shows photographs and quantitative results of the *in vitro* treatment efficacy of US without and with LY-shelled MBs against *P. acnes* colonies (n = 6). At the same concentration of LY, the growth of *P. acnes* was inhibited by 86.08 ± 2.99% in the LY-shelled MBs group, and more effectively than in the LY solution group (group LY) (57.74 ± 3.09%) (p < 0.001). US sonication at various acoustic power densities (1, 2, and 3 W/cm^2^) did not markedly suppress the growth of *P. acnes* (16.80 ± 5.94%, 24.76 ± 2.88%, and 34.66 ± 2.36%, respectively). However, combining US with LY-shelled MBs greatly suppressed the growth of *P. acnes*, by 95.79 ± 3.30%, 97.99 ± 1.16%, and 98.69 ± 1.13% for acoustic power densities of 1, 2, and 3 W/cm^2^, respectively. The results showed that combining LY-shelled MBs with US can markedly inhibit the growth of *P. acnes*, but with the effect not varying with the US power density.

### Effects of LY-shelled MBs combined with US on P.-acnes-induced inflammatory skin disease

**Figure 6:**
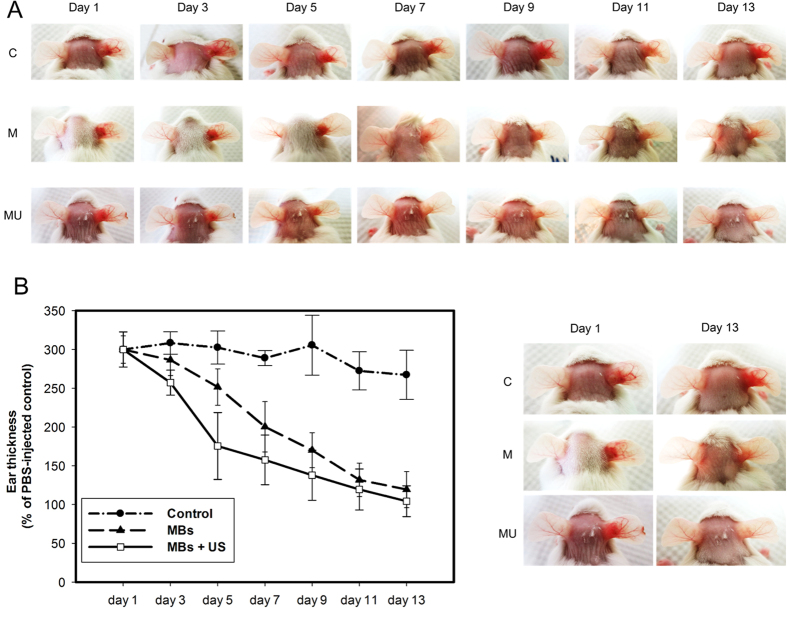
(**A**) Significant ear inflammatory reactions (right ears) were observed 24 h after the *P. acnes* injection. Representative images from each treatment group at various times were shown. (**B**) Quantification of ear thickness. Untreated and uninfected ears (left ears) served as a negative control.

To investigate the therapeutic effects of LY-shelled MBs combined with US against *P.-acnes*-induced inflammatory skin disease, *P. acnes* was injected intradermally into the right ears of ICR mice (n = 6). After the injection, LY-shelled MBs and LY-shelled MBs combined with US were applied to the surface of the skin of the right ear. As shown in Fig. 6A, significant ear inflammatory reactions were observed 24 h after the *P. acnes* injection. Figure 6B shows that during the first 7 days, the ear thickness was reduced twofold for LY-shelled MBs treatment with US relative to the control group (*p* < 0.01). During the first 5 days, the ear thickness was reduced 1.45 fold for LY-shelled MBs treatment with US relative to the LY-shelled MBs treatment alone group (p < 0.05). After 13 days of treatment, the LY-shelled-MBs-treated ears showed noticeably reduced ear inflammatory reactions. In particular, LY-shelled MBs and LY-shelled MBs combined with US resulted in a threefold reduction of ear thickness compared to the ears injected only with living *P. acnes* (Fig. 6B). At that time there were no inflammatory reactions observed in the group treated with LY-shelled MBs and US.

### Histochemistry

**Figure 7:**
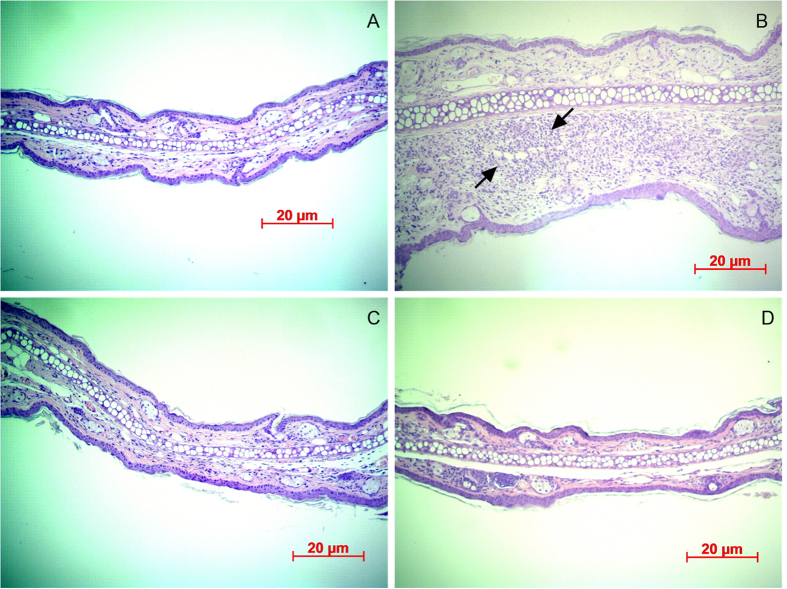
Histological observations of ears of ICR mice with hematoxylin and eosin staining. (**A**) Original ear. (**B**) Increases were observed in both the ear thickness and the number of infiltrating inflammatory cells (arrows) surrounding the injection site of *P. acnes*. The ear thickness, swelling, erythema, and inflammatory reactions were reduced (**C**) in LY-shelled-MBs-treated ears and (**D**) in ears treated with both US and LY-shelled MBs.

Histological observations revealed that the *P. acnes* injection induced a considerable increase in the number of infiltrated inflammatory cells (Fig. 7B). As shown in Fig. 7B, significant ear swelling, redness, and erythema were observed 24 h after the *P. acnes* injection. After 13 days of treatment there were noticeable reductions in ear thickness, swelling, erythema, and inflammatory reactions in the LY-shelled-MBs-treated ears and those ears also treated with US (Fig. 7C and D).

## Discussion

Previous studies have found that the mean diameter and the size distribution of MBs vary with the length of the sonication and denaturation times, with a shorter sonication and/or denaturation time yielding larger MBs. Denaturation from 2 to 5 min coupled to 30 s of sonication reportedly produced an optimal size distribution^23^. In that study, the optimal conditions for producing consistent and suitably sized MBs (2–3 μm) were sonication for 20–30 s at powers of 40–80 W. The MBs formed when using higher amplitude (>50%) and longer times (>30 s) were fragmented, which was probably due to strong shear forces breaking the MBs and generating cross-linked protein fragments^24^. Moreover, it was observed that increasing the concentration of the reducing agent (DL-DTT) yielded smaller MBs with a narrower size distribution^25^. In accordance with the above results, in our study the size distribution did not vary for different sonicator powers, with LY-shelled MBs constructed using sonicator powers of 80 and 120 W exhibiting a narrow size distribution ranging from 1500–4200 nm in diameter using the same concentration of DL-DTT.

The concentration of LY-shelled MBs increased with the sonicator power. Figure 4 shows that the antimicrobial efficacies of LY-shelled MBs constructed using sonicator powers of 120 and 180 W were significantly higher than for 80 W at each concentration (*p* < 0.05). LY-shelled MBs constructed using a sonicator power of 120 W had a more uniform distribution, indicating that combining LY-shelled MB in this condition can improve the US sensitivity and significantly enhance the antimicrobial efficacy^26^. In this study, the antimicrobial efficacy was the most stable for 5% LY-shelled MBs constructed using a sonicator power of 120 W, and hence these conditions were used in all of the experiments.

Figure 5 shows that *P. acnes* was inhibited more effectively in the LY-shelled MBs group than in the LY solution group for LY at the same concentration. This may be due to phagocytosis of the MBs increasing the cytotoxicity. Previous cytotoxicity tests indicated that phagocytosis of the MBs by macrophages starts within 6–8 hours, with both single MBs and clusters of MBs observed in the cytoplasm of the cells^27^. US sonication at various power densities can suppress the growth of *P. acnes*, but the changes were not statistically significant. While the suppression of *P. acnes* growth increased mildly by increasing the power density of US, combining LY-shelled MBs combined with US could significantly inhibit the growth of *P. acnes*. The magnitude of the inhibition did not depend on the US power density (1–3 W/cm^2^), which represents evidence that the antimicrobial efficacy depends more on US-mediated MBs cavitation than on the US power density.

In the animal experiments, the recovery rate for the ear in Group MU at day 5 was 62%, reaching 71% at day 7 and 98% at day 13; the corresponding rates in Group M were 24%, 49%, and 90%, respectively. The histological observations revealed only mild increases in ear thickness and inflammatory reactions were observed in Group M. Although the ear thickness of LY-shelled MBs treatment with US is similar to LY-shelled MBs alone at day 13, mild inflammatory reactions still can be observed in LY-shelled MBs alone group in histological observation. These results confirmed that the combined treatment with US and LY-shelled MBs can reduce the treatment duration, inhibit *P.-acnes*-induced inflammation, and significantly improve the prognosis of skin disease. In addition, some new types of drug, gold nanoparticles, immobilized on LY-shelled MBs could be created to improve the antimicrobial efficacy^28^. LY-triclosan complexes also could be a candidate to be produced in the form of MBs to enhance the treatment effects.

## Conclusion

This study investigated a new treatment model for antibacterial effects on *P. acnes* using LY-shelled MBs and US-mediated LY-shelled MBs cavitation. It was found that increasing the sonicator power increased the concentration of LY-shelled MBs but did not change the size distribution of the produced MBs. The antimicrobial efficacy of 5% LY-shelled MBs constructed using a sonicator power of 120 W was more stable and hence selected for all of the experiments. In the *in vitro* experiments, LY-shelled MBs combined with US significantly inhibited the growth of *P. acnes*, with this effect not depending on the US power density. In the *in vivo* experiments, combined treatments of US and LY-shelled MBs reduced the treatment duration, inhibited *P.-acnes*-induced inflammation, and significantly improved the prognosis of skin disease.

## Additional Information

**How to cite this article**: Liao, A.-H. *et al*. Treatment effects of lysozyme-shelled microbubbles and ultrasound in inflammatory skin disease. *Sci. Rep.* **7**, 41325; doi: 10.1038/srep41325 (2017).

**Publisher's note:** Springer Nature remains neutral with regard to jurisdictional claims in published maps and institutional affiliations.
